# Diseases of the Respiratory Organs

**Published:** 1905-03-11

**Authors:** 


### DISEASES OF THE RESPIRATORY ORGANS.

The discussion on what are the exact constituents of foul air which are injurious to health was started by Haldane's paper at Oxford, and in view of the popular belief at the present time in the value of fresh air, not only for phthisical patients, but even for the healthy, there appears an extraordinary difference of opinion and a curious absence of scientific facts. Louis Parkes[1] confesses that the increased $CO_2$ is not of itself injurious. Nature never allows us to take a single breath of air pure from $CO_2$ and other contaminations. There is always about 600 volumes per 10,000 of $CO_2$ in the pulmonary alveoli, and an increase at once induces an increased respiratory action, so that the percentage remains constant. While the quantity in pure air is 4 parts in 10,000, it rarely rises above 50 in excessively crowded rooms, and no poisonous effects are found till the quantity in the surrounding air reaches 500 in 10,000 volumes. The diminution of oxygen in crowded places does not appear ever to reach that found in healthy places at high altitudes. Ozone, again, is not certainly known to exist in the atmosphere at all. Organic matters contained in expired air were formerly supposed to be important. No volatile organic poison seems to be present in expired air, and there is no proof that the ammonia derived from washing expired air is derived from the lungs. It may arise from particles shed from the dirty skins and foul teeth and mouths of a crowd. Unpleasant odours given off from a crowd are doubtless due to various little-known volatile substances, and may possibly by their action on the olfactory nerves induce reflex changes in the body. The heat and moisture of the air in crowded rooms is not so great as that artificially produced in other situations. Micro-organisms of certain diseases, such as phthisis, are, of course, scattered in the air by coughing and talking; but, apart from these definite pathogenic bacteria, which propagate their own diseases, the presence of other germs in the air is not of importance.

It is probable that there is some constituent of air which has been vitiated by human respiration which has an injurious action upon health, but it is quite unknown what this is. In defining satisfactory ventilation in factories we are compelled to take a purely arbitrary standard, the amount of $CO_2$. Thus nine volumes has been ordered as the limit for moist rooms in cotton factories. Haldane and Osborne propose 12 volumes by day for factories generally; but serious objection has been made to this, as actually higher than the existing average, viz., 10 volumes.

**Coal-gas Poisoning.**—In papers by Gilman Thompson and Hubbard [2] the importance of bleeding and saline injections is insisted on. Thompson's paper is founded on a study of 90 cases, of which 17 were fatal. He thinks that the symptoms are produced by some specific action of the gas on the nervous system and not by the mere deoxydising power of the CO. Leucocytosis is generally present, and in fatal cases exceeds 18,000, reaching even to 50,000. The polynuclear cells are in excess. In nearly all cases pyrexia is present, and is frequently preceded and followed by subnormal temperatures. Coma bears no relation to the intensity of the fever, and may last four or five days even in patients who recover. Convulsions and various brain lesions are common, and may be a cause of death. It is curious that broncho-pneumonia and other pulmonary lesions are by no means constantly present.

**Fibroid Phthisis.**—A. James [3] remarks that fibrotic changes will differ according as they take place in a yielding organ like the liver, or in one enclosed in an unyielding case like the lungs, with air vesicles under considerable pressure. Hence the double appearance of fibrosis and emphysema in this disease. Though many cases are due to chronic bronchitis, irritating dust, or syphilis, there are others in which no cause can be found except a low degree of nutrition—the so-called fibroid diathesis—which hinders recovery of the tissues after some slight attack of disease. It is, however, remarkable that tubercle bacilli are usually absent. As to treatment, improved nutrition, with cod-liver oil and the syrups of iodide and phosphate of iron, may check the fibrotic degeneration.

**The Early Diagnosis of Tuberculosis.**—T. W. Price [4] refers to the fact that a diagnosis of phthisis can often be made weeks, and even months, before bacilli occur in the sputum. On the other hand, in many old people with "winter cough," we are apt to overlook phthisis for want of testing the sputa. Among early signs, an evening pyrexia, dyspepsia, loss of weight even without a cough, or a slight pleurisy, or an attack of what is called "influenza," may lead to an examination and the detection of phthisis. Again, in some cases, chronic bronchitis or slight hæmoptysis may be the first symptom. The first physical signs are most often met with about $1\frac{1}{2}$ inch below the summit of the lung towards the posterior and external borders, and then downwards and forwards. Or they may be found in the outer part of the second and third spaces, and next in the upper part of the axilla. If with these there are found signs on the same side midway between the fifth dorsal spine and the border of the scapula, the diagnosis is almost certain. Deficient expansion of one apex is often seen before any alteration occurs in the percussion note. Any absence of the normal excess of vocal fremitus at the right apex should be carefully noted. In percussion we may be led astray by scoliosis or great muscular development on one side. Prolonged expiratory sounds at the apex, if low-pitched and blowing, are often due to emphysema; if high and bronchial, to consolidation. Feeble breath sounds where harsh ones normally occur are significant. At first there are no râles, then we find small crackles, chiefly inspiratory, then clicking sounds, and later on larger and more liquid ones. If these râles persist when the patient coughs or takes a long breath, and are strictly localised near the apices, we can certainly diagnose phthisis. To exclude false râles from the larynx the patient should not swallow after coughing, and generally we should take care not to make our diagnosis from a single physical sign. Professor Cornet [5] holds that percussion is of little value in the early stages of the disease, because a consolidated patch, to produce a change in the note, must be at least four to six centimetres in diameter and two centimetres deep. The same writer points out the bacillus is chiefly to be found in places where uncleanly consumptives live, and only rarely elsewhere. Hence the chance of infection in the streets is much over-estimated. W. T. Gairdner [6] emphasises the importance in examination by percussion of minimising the stroke in most cases when we wish to delimit the area of subjacent organs. Deep percussion is necessarily inexact; what is gained in the volume of sound is lost in definiteness. Hence the deep percussion of Piorry is not to be relied on.

**Treatment.**—The value of the synthetic cinnaminate of soda, called Hetol, when given intravenously, is discussed by T. Barrett Heggs,[7] who finds it a useful adjunct to other treatment, though not a specific. Injections were given by him on alternate days, and rapidly increased up to 20, 30, or even 50 milligrammes, with antiseptic precautions. Two cases were treated for six mouths and five for six weeks. All improved, and all but one gained weight. In one case this was as much as $10\frac{1}{8}$ lbs. A definite leucocytosis followed each injection—the maximum was generally obtained with about 20 milligrammes. No complete cure was obtained. A. E. Wright [8] lays down that we may kill off bacteria in the body either by introducing antiseptic substances or by protective substances produced by the organism. The former method has especially failed in tuberculosis. To employ the second method, by introducing vaccines, we must note that a negative phase follows each injection, during which the blood contains less than usual of the natural protective. Then follows a phase in which there is a large excess, followed by a slight permanent one. If we carefully regulate excessive doses and the time of administration, we can obtain an accumulation of increased resistances, but if the doses are ill-judged or wrongly timed we may lower the protective resistance. The vaccine which causes the production of a "tubercular tropic" or anti-bacterial substance in the body may be made from a sterilised culture of the bacillus, such as the T. R. tuberculin or from a sterilised suspension of bacilli of exactly measured strength. The tubercular patient had probably originally a low degree of resistance, and has failed to react to the infection which assailed him. We have to increase the richness of the blood in protectives to prevent the spread of the disease, and secondly to attack the nidus by sending against it a stream of lymph similarly enriched.

[1] Pract., Oct. [2] Med. Rec., July, Sept. [3] Scottish Med. and Surg. Jour., Sept. [4] Edin. Med. Jour., Oct. [5] Brit. Med. Jour., Nov. 12. [6] Edin. Med. Jour., Nov. [7] Lancet, Oct. 22. [8] Clin. Jour., Nov. 9.

